# The effect of increasing the sulfation level of chondroitin sulfate on anticoagulant specific activity and activation of the kinin system

**DOI:** 10.1371/journal.pone.0193482

**Published:** 2018-03-01

**Authors:** J. Hogwood, A. Naggi, G. Torri, C. Page, P. Rigsby, B. Mulloy, E. Gray

**Affiliations:** 1 National Institute for Biological Standards and Control, Blanche Lane, Herts, United Kingdom; 2 Sacker Institute of Pulmonary Pharmacology, Institute of Pharmaceutical Science, King’s College London, United Kingdom; 3 Institute for Chemical and Biochemical Research ‘‘G. Ronzoni”, Milan, Italy; Institut d'Investigacions Biomediques de Barcelona, SPAIN

## Abstract

Oversulfated chondroitin sulfate (OSCS) was identified as a contaminant in certain heparin preparations as the cause of adverse reactions in patients. OSCS was found to possess both plasma anticoagulant activity and the ability to activate prekallikrein to kallikrein. Differentially sulfated chondroitin sulfates were prepared by synthetic modification of chondroitin sulfate and were compared to the activity of OSCS purified from contaminated heparin. Whilst chondroitin sulfate was found to have minimal anticoagulant activity, increasing sulfation levels produced an anticoagulant response which we directly show for the first time is mediated through heparin cofactor II. However, the tetra-sulfated preparations did not possess any higher anticoagulant activity than several tri-sulfated variants, and also had lower heparin cofactor II mediated activity. Activation of prekallikrein was concentration dependent for all samples, and broadly increased with the degree of sulfation, though the di-sulfated preparation was able to form more kallikrein than some of the tri-sulfated preparations. The ability of the samples to activate the kinin system, as measured by bradykinin, was observed to be through kallikrein generation. These results show that whilst an increase in sulfation of chondroitin sulfate did cause an increase in anticoagulant activity and activation of the kinin system, there may be subtler structural interactions other than sulfation at play given the different responses observed.

## Introduction

In 2008 there were many adverse events, including fatalities, associated with the administration of certain preparations of heparin [1]. Analysis of the heparin preparations associated with these events found a contaminating material, oversulfated chondroitin sulfate (OSCS) [2]. This contaminating material was found to interact with the contact system in plasma through activation of prekallikrein [3] which cleaved high molecular weight kininogen, leading to the formation of bradykinin [4]. Bradykinin is a potent vasodilator which causes a hypotensive response, and has been proposed to be the most likely cause of death in numerous patients who were infused with contaminated heparin preparations [5]. Animal studies using rats and pigs have shown that OSCS can cause a marked drop in blood pressure that could be prevented by administration of a bradykinin receptor antagonist [3, 5, 6]. This indicates that activation of the kinin system leading to generation of bradykinin is the likely mechanism of action of the adverse effects seen with OSCS contaminated heparin.

Structural analysis of OSCS purified from contaminated heparin preparations showed that chondroitin sulfate (CS) had been subjected to chemical oversulfation to increase the number of sulfates to four on the main disaccharide unit [2]. Furthermore, detailed characterization showed that OSCS has *O*-sulfates at the 4 and 6 positions on N-acetylgalactosamine, and positions 2 and 3 on glucuronic acid [2]. This contrasts with native chondroitin-4- sulfate and chondroitin-6-sulfate which are predominantly sulfated only at position 4 or position 6 of *N*-acetylgalactosamine respectively [2, 6]. An increase in the degree of sulfation can increase the anticoagulant activity of chondroitin sulfate [7, 8] and the anticoagulant effect was observed in normal human plasma, and plasma depleted of antithrombin [9]. This is in agreement with previous reports that highly sulfated chondroitin sulfates [10, 11] have an anticoagulant action in plasma and it has been proposed to be primarily through potentiation of heparin cofactor II (HCII). The limitation of all these studies is the use of imprecise assay systems from which anticoagulant activity through HCII is inferred, and the assumption that binding interaction between OSCS and HCII indicates the potentiation of HCII inhibition of thrombin. The assumption that anticoagulant activity is solely through HCII [9] in antithrombin deficient plasma ignores other known mechanisms which heparin, and therefore perhaps OSCS, can act through, and these include potentiation of tissue factor pathway inhibitor on factor Xa [12] or protein C inhibitor on thrombin and factor Xa [13].

The current study aims were to determine: the specific activities of differentially sulfated chondroitin sulfates by multiple anticoagulant methods, with a particular focus on heparin cofactor II activity; to investigate the correlation between the degree and sulfation pattern with anticoagulant activity of OSCS; and to measure the effect that increasing the sulfation of chondroitin sulfates has on prekallikrein activation and whether generation of bradykinin by OSCS is associated with production of kallikrein.

## Materials and methods

### Materials

Synthetic oversulfated chondroitin sulfates were prepared from chondroitin sulfate A (Sigma Aldrich, Italy) as previously described by use of pyridine–sulfur trioxide complex in dimethylformamide [2]. The sample of OSCS isolated from contaminated heparin was a gift from Leo Pharma (Cork, Ireland). Chrondroitin-4- sulfate (from bovine trachea) and dextran sulfate (from *Leuconostoc* spp.) were obtained from Sigma, UK. The 5^th^ International Standard for Unfractionated Heparin, 97/578, human thrombin and human antithrombin preparations for potency assays and a separate antithrombin for the fluorescence titration assay were from NIBSC (Potters Bar, UK). Sheep and human plasma for clotting assays were purchased from First Link, UK and National Blood Transfusion Service, UK, respectively. Activated Partial Thromboplastin Time (APTT) reagents were Actin-FS from Sysmex, UK and APTT-SP from Werfen, UK. Bovine activated factor X was from Diagnostic Reagents, UK. Human heparin cofactor II was from Enzyme Research, UK. Chromogenic substrates S2302, S2238 and S2765 were from Werfen, UK. Pooled normal human plasma for prekallikrein and bradykinin assays was obtained from George King, Kansas USA. The Bradykinin ELISA kit was obtained from Enzo Research, Exeter, UK. All other reagents were analytical grade and obtained from Sigma, UK.

### Methods

#### NMR experimental procedure

Proton and HSQC-NMR spectra were obtained at 500 MHz with a Bruker Avance 500 spectrometer equipped with a 5-mm TXI probe. Chemical shift values were measured downfield from trimethylsilyl propionate sodium salt (TSP) as standard at 40°C and the offset was set at the residual water resonance. Samples (10 mg) were previously submitted to a double lyophilisation from D_2_O and finally dissolved in 0.6 ml of deuterium oxide (99.996 at% D_2_O). Mono-dimensional ^1^H spectra were obtained with pre-saturation of residual HDO, 128 scans, and a recycle delay of 10 s. Two-dimensional gradient enhanced HSQC-NMR spectra were recorded via double inept transfer with a carbon decoupling (garp4) during acquisition using a sensitivity improvement trim pulses in inept transfer and shaped pulses for all 180° pulses on ^13^C channel (hsqcetgsisp2.2 Bruker sequence) with 512 increments of 64 scans each. The polarization transfer delay (D = 1/[2 ^1^J_C–H_]) was set with ^1^J_C–H_ coupling values of 139–150–170 Hz. The matrix size 1K x 512 was zero filled to 4K x 2K by application of a squared cosine function prior to Fourier transformation. Integration of cross-peaks was made using standard Bruker XWINNMR 3.5 software.

#### Degree of sulfation

Integration of heteronuclear single quantum coherence (HSQC-NMR) spectra was validated for determination of compositional analysis of heparins and applied to chemically modified glycosaminoglycan samples. The relative molar percentages of 6-OSO_3_, 4-OSO_3_ groups for galactose amine residues and of non-sulfated, 2,3-OSO_3_, 2-OSO_3_, and 3-OSO_3_ groups for glucuronic acid residues was evaluated from the integration of the corresponding volumes of their anomeric signals (see Table A and Fig A in S1 File).

#### Molecular weight determination

Molecular weight determinations were performed by HPSEC- TDA on a Viscotek (Houston, Texas) instrument equipped with a VE1121 pump, Rheodyne valve (100 μl), and triple detector array 302 equipped with refraction index (RI), viscometer, and light-scattering (90° and 7°) systems [14] for synthetic OSCS samples only. Molecular weights for all samples were also determined by the USP broad standard method for molecular weight calibration of Unfractionated Heparin, adapted from the USP monograph for Heparin Sodium [15].

#### Anticoagulant assays

The test samples were assayed against the 5^th^ International Standard for unfractionated heparin using an automated coagulometer (ACL-TOP 500, Instrumentation Laboratories, UK). Concentration response curves, with at least 3 dilutions and replicates for both standard and test were assayed within each run. Potency estimates and statistical analysis of data were carried out in accordance with European Pharmacopoeia guidelines [16], using CombiStats 5.0 (European Directorate of the Quality of Medicine, (EDQM), France). Apart from the human plasma assay which was analysed using a slope ratio model, all other assays were analysed using a parallel line bioassay model. These analysis methods use multiple dilutions which estimate the activity of a sample relative to the standard used and allow for an assessment of model validity by ANOVA i.e. linearity and common intercept for slope ratio model, linearity and parallelism for the parallel line model. This is the recommended method of potency assignment according to EDQM guidelines (16). The specific activity in IU/mg was calculated from the potency obtained by each assay method relative to the dry weight of the test sample.

Sheep-plasma assay: A modified version of the previous European Pharmacopeia assay [17] based on the APTT, using sheep plasma was carried out. This method was adapted to be carried out on an automated coagulometer. Briefly, samples were prepared in tris-buffered saline (TRIS 50mM, NaCl 150mM, pH 7.4) and each dilution was mixed with sheep plasma before addition of Actin-FS, the APTT reagent. After incubation, clotting was started by addition of calcium chloride and the clotting time recorded.

Human-plasma assay: This plasma based APTT assay was performed in a similar manner as the sheep plasma assay. The samples were diluted, mixed with human plasma and clotting times were recorded following the addition of APTT-SP (APTT reagent), and calcium chloride as recommended by the manufacturer.

Anti-Xa and anti-IIa assays: The methods used were modified from the method as described in the United States Pharmacopoeia (USP) general monograph for Assay of Heparin (USP Monograph, General Chapter 208). The methods were modified for use on an automated coagulometer.

Heparin cofactor II assay: Samples were diluted with tris buffered saline, and mixed 1-to-1 with heparin cofactor II (2.5μg/ml). Human thrombin (1 IU/ml) was added to the mixture and incubated for 420 seconds prior to addition of chromogenic substrate specific for thrombin (S2238, 1mM). The change in colour was recorded at 405nm.

#### Kallikrein generation assay

The test compounds were dissolved in distilled water and a dilution series was made using a low salt buffer (50mM TRIS, 14mM NaCl pH 7.8). Each sample was mixed with an equal amount of pooled normal plasma prior to incubation at 37°C. The blank control with pooled normal plasma was found to give no kallikrein activity. Experiments found that a five-minute incubation between sample and plasma provided maximal kallikrein formation (data shown in Table B in S1 File). The samples were then incubated for five minutes, prior to addition of chromogenic substrate (S2302, 0.5mM) and colour was developed for four minutes before stopping the assay using 50% acetic acid. The reaction samples were read at 405nm with a 490nm reference reading subtracted. The amount of kallikrein generated is proportional to the amount of chromogen yielded. Due to the absence of reference materials for this assay, dextran sulfate which is known for its ability to cause the activation of prekallikrein [18] was used as a positive control.

#### Bradykinin generation assay

Samples were prepared as described in the kallikrein generation assay. Following the five minute incubation time between test compound and normal pooled plasma, samples were diluted 10 fold with ice cold ethanol (as described in the commercial Bradykinin ELISA kit, Enzo Research, Exeter, UK) and stored on melting ice for 1 hour [4]. Samples were centrifuged at 10,000g for 5 minutes, the supernatant was collected and dried using a rotary evaporator and stored -40°C. Bradykinin was measured following the method as described in the commercial ELISA Kit.

#### Antithrombin titration assay

Measurement of the ability of each oversulfated chondroitin sulfate material to bind to antithrombin was assessed using an antithrombin titration assay [19]. Briefly, a concentration range 5 to 100μg/ml of each sample was prepared in buffer (50mM TRIS, 150nM NaCl, 10 mM EDTA, 0.05% Tween-20, pH 7.4). The concentrations for each sample (100μl) were mixed with 100μl purified antithrombin (NIBSC, South Mimms UK) and the increase in fluorescence (λ_EX_ 280 nm, λ_EM_ 340 nm) over antithrombin alone was recorded.

## Results

### Physicochemical analysis

Six differentially sulfated samples of CS were prepared. Table 1 shows the sulfation patterns of different preparations of over sulfated chondroitin sulfates. The degree of sulfation ranged from 2.4 to 4.0. Percentage distribution of sulfate groups per residue was evaluated by ^1^H/^13^C NMR correlation experiments. Spectra with assignments are reported in supplementary material (Fig A and Fig B in S1 File). In all cases, with the exception of the disulfated chondroitin sulfate with 90% GalNAc_6S_, the galactosamine was fully sulfated by comparison with native CS (Table 1). For the tri-sulfate and tetra-sulfate samples the further increase in overall sulfation was at glucuronic acid carbon-2 and carbon-3. For the three tri-sulfated samples (3.0, 3.1 and 3.2 sulfates), the key difference is more carbon-2 and -3 in the glucuronic acid being sulfated (25%, 28% and 35%).

**Table 1:** Sulfation pattern (%) of different preparations of OSCS.

	Glucuronic Acid				Galactosamine	
Disaccharide Sulfation degree	G_2S,3S_	G_2S_	G_3S_	G	GalNAc_4S_	GalNAc_6S_
CS 1.0 (native CS)	0	0	0	100	70	30
CS 2.4	18	5	10	67	100	90
CS 3.0	25	41	12	22	100	100
CS 3.1	28	42	12	17	100	100
CS 3.2	35	42	12	11	100	100
CS 4.0	100	0	0	0	100	100
CS 4.0 #2	100	0	0	0	100	100

Level of sulfation indicated for each sample on carbon 2 and/or 3 in glucuronic acid, and on carbon 4 and 6 in galactosamine via NMR HSQC quantitative analysis.

The molecular weights were measured by two different methods. The first method used a triple detector array (TDA) calibrated with samples of known weight average molecular weight (Mw), polydispersity and intrinsic viscosity [14], and the second method used a broad standard calibrant (the USP Heparin Sodium Molecular Weight Calibrant, F0L483), an unfractionated heparin sample of defined molecular weight distribution [15]. The two different analytical approaches obtained different number average (Mn) and weight average (Mw) molecular weights, with the TDA calculating higher Mn and Mw than the broad standard method (Table 2).

**Table 2:** Molecular weights of additionally sulfated CSs. Molecular weights have been measured via HP-SEC/ TDA.

	Via HP-SEC/TDA			USP Broad Standard Method		
	Mn	Mw	PD	Mn	Mw	PD
**CS 2.4**	13400	31100	1.6	12680	17600	1.4
**CS 3.0**	19900	28000	1.4	14980	21050	1.4
**CS 3.1**	21780	28560	1.3	16280	21960	1.4
**CS 3.2**	18400	23800	1.3	15860	21390	1.4
**CS 4.0**	29200	35100	1.2	16360	21100	1.3
**CS 4.0 #2**	30100	35700	1.2	16820	21750	1.3
**Isolated OSCS**	-	-	-	11480	15740	1.4

For the purified OSCS sample the molecular weight was calibrated against USP Heparin Sodium Molecular Weight Calibrant (F0L483). Mn = number average molecular weight; Mw = weight average molecular weight; PD = Polydispersity (Mw/Mn).

The molecular weights of the preparations were measured and compared to OSCS purified from contaminated heparin preparations against the USP Heparin Sodium Molecular Weight Calibrant (Table 2). All the semi-synthetically prepared samples had molecular weights (Mw) higher than purified OSCS (Mw range 17600 to 21960 compared to 15740); however, the polydispersity was broadly comparable for all samples (range 1.3 to 1.4).

### Anticoagulant assays

Table 3 shows the anticoagulant activity of all the OSCS samples and a unadulterated heparin sample relative to the 5th IS for unfractionated heparin. Apart from the CS-A sample, all samples gave statistically valid results by slope ratio analysis in the human plasma assay and by parallel line analysis for all other assays. Validity assessment was by ANOVA [16] using CombiStats which was considered to confirm that samples gave a linear dose response across the doses used, had a common intercept in the by slope ratio model or parallel dose-response relationships in the parallel line model. The heparin sample included gave anticoagulant activity values which were similar across all the methods used. Native CS had no measurable anticoagulant activity in either plasma based or anti-Xa and anti-IIa assays. Specific activity for the samples by the anti-Xa and anti-IIa assays were all below 5 IU/mg, indicating that these samples could not potentiate the inhibitory action of antithrombin to a high degree. Both synthetic and purified oversulfated CSs expressed relatively high anticoagulant activity in plasma based clotting assays. With the exception of CS2.4 which gave similar activity in both types of assay, all other OSCS showed 3–8-fold higher activity in the sheep plasma assay than in the human plasma method. There was no apparent correlation between degree of sulfation and the anticoagulant activity in plasma-based assays. In the heparin cofactor II mediated assay, the 3 tri-sulfated CSs gave three-fold higher activities than the tetra- and di-sulfated CSs, showing that an increase in sulfation would not necessarily result in higher activity. Overall, the synthetic tetra-sulfated material (CS4.0) has a very similar anticoagulant profile to the purified OSCS.

**Table 3:** Anticoagulant activities of chemically sulfated CSs and purified OSCS from contaminated heparin.

	IU/mg (95% Confidence Limits)				
sulfate level / disaccharide	Human APTT	Sheep APTT	Anti-Xa	Anti-IIa	HCII
CS 2.4	16 (15–17)	41 (40–42)	<1	<1	152 (134–173)
CS 3.0	52 (50–54)	129 (126–132)	1.4 (1.2–1.6)	3.1 (2.9–3.3)	753 (662–858)
CS 3.1	55 (54–56)	141 (137–144)	1.6 (1.4–1.8)	4.2 (3.9–4.5)	906 (796–1032)
CS 3.2	52 (50–54)	138 (126–132)	2.0 (1.8–2.2)	3.7 (3.3–4.3)	898 (788–1022)
CS 4.0	48 (47–49)	137 (134–140)	5.3 (4.8–5.9)	4.5 (3.9–5.3)	324 (259–404)
CS 4.0 (#2)	50 (49–51)	142 (139–145)	5.9 (5.3–6.6)	3.8 (3.5–4.0)	264 (212–330)
Purified OSCS from Contaminated Heparin	56 (54–57)	181 (174–189)	5.7 (5.2–6.3)	4.9 (4.7–5.1)	187 (166–210)
Chondroitin 4 sulfate	<1	<1	<1	<1	1.9 (1.8–2.1)
Heparin sample	209 (191–229)	226 (219–233)	214 (207–221)	209 (196–223)	241 (223–261)

Values are IU/mg estimated against the 5th International Standard for unfractionated heparin (NIBSC, 97/578), with 95% confidence limits in brackets. All results were considered valid using multiple dilution models–slope ratio for plasma (human and sheep) assays and parallel line for purified protein (antithrombin, anti-IIa, anti-IIa, HCII anti-IIa) assays.

Samples were all incubated with thrombin or factor Xa only and found to have no direct inhibitory action on either enzyme with results similar to buffer only.

### Kallikrein generation

**Fig 1:**
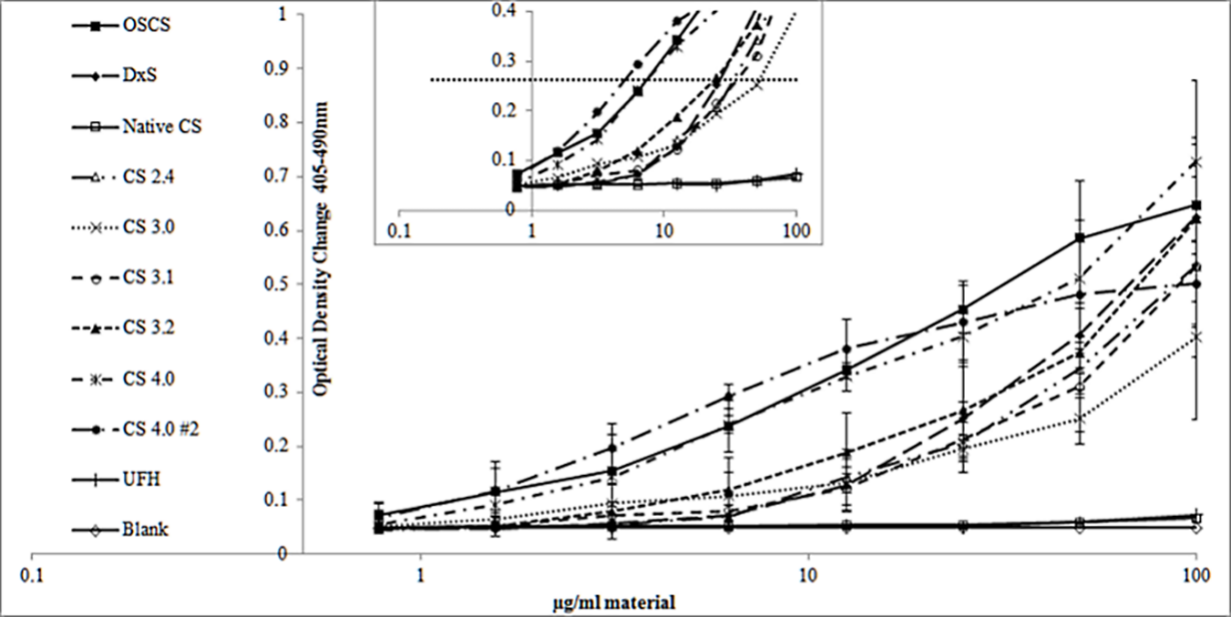
Kallikrein generation in normal pooled plasma by synthetic and purified OSCSs, native chondroitin sulfate, dextran sulfate and unfractionated heparin. Error bars = standard deviations (SD), n = 6, note lines for native CS, unfractionated heparin and plasma alone are overlapping. Inset chart shows line (**….**) indicating Δ0.2 OD above baseline used to select concentration of samples for the bradykinin assay.

Fig 1 summarises the concentration dependency of kallikrein generation for all the samples. Both native CS and unfractionated heparin did not generate kallikrein at any of the concentrations tested, with results very similar to plasma alone. In contrast, all over-sulfated CSs were able to generate kallikrein, in a concentration dependent manner. The di- and tri-sulfated CSs gave a similar concentration response profile, whilst the synthetic tetra CS and the purified OSCS exhibited higher activity especially at lower concentrations (<20μg/ml).

### Bradykinin generation

**Fig 2:**
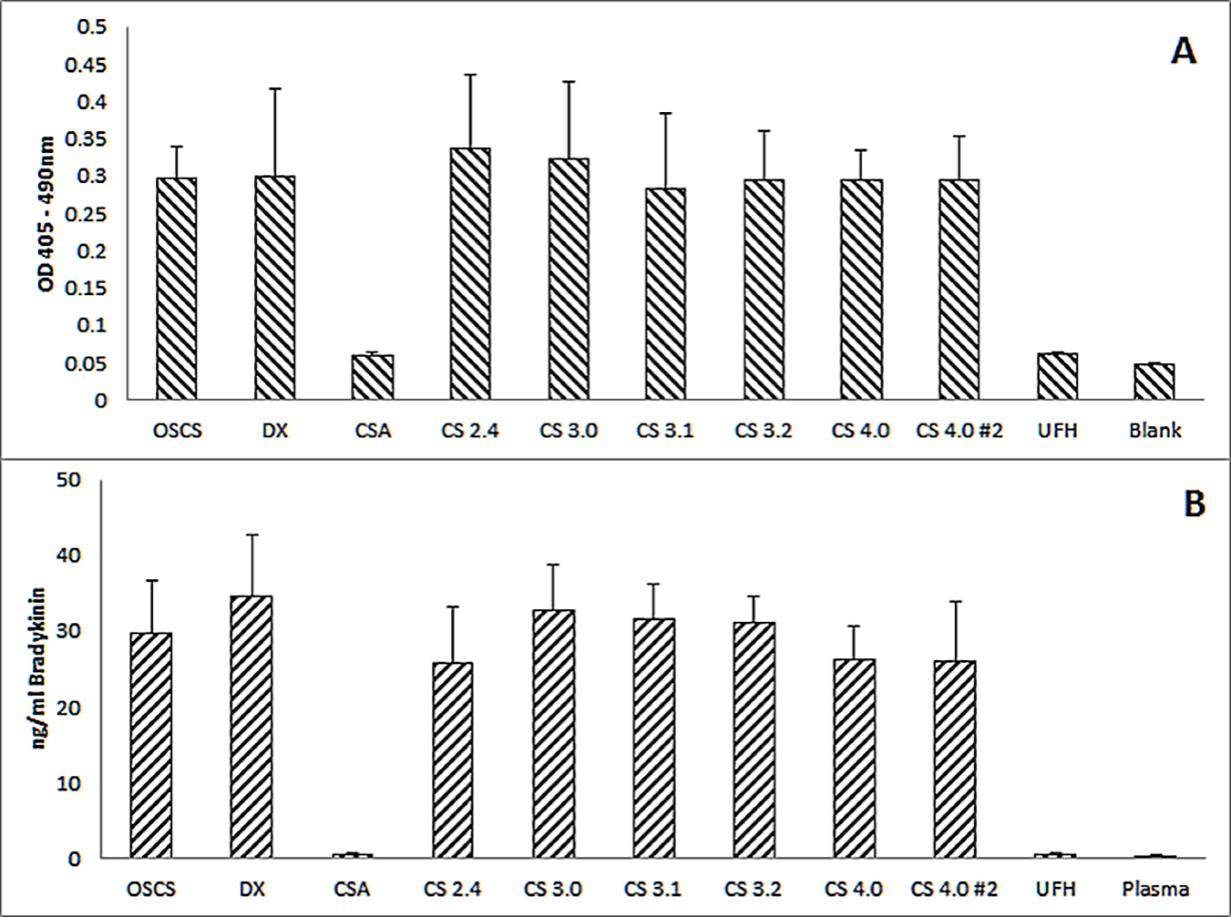
Kallikrein and Bradykinin levels obtained from a defined quantity of synthetic and purified OSCS, native chondroitin sulfate, dextran sulfate and unfractionated heparin. Different amounts of each material, predicted to generate the same level of **(A)** kallikrein as determined by optical density increase and **(B)** bradykinin, were used (see Table 4). No statistical difference was observed between the samples (oneway ANOVA, p = 0.883 and 0.568 respectively). CS-A and UFH were included at 100μg/ml, the highest level used. Error bars = standard deviation; n = 6.

It has been shown that OSCS can lead to the generation of bradykinin [4], but it has not been shown that OSCS triggered kallikrein activity leads to bradykinin production. While it is reasonable to assume that this mechanism will lead to some bradykinin production, it is possible that the OSCS samples might also induce bradykinin through other, non-kallikrein associated routes. To investigate the link between kallikrein and bradykinin production a single concentration of each sample was used. A concentration which generated a 0.2 optical density change (See Fig 1, inset) in the kallikrein assay was chosen (Table 4). On the basis of these concentrations, the samples each generated the same amount of kallikrein (Fig 2A) and also the same amount of bradykinin (Fig 2B) as each other. As with the kallikrein generation assay, native CSA and unfractionated heparin at 100μg/ml did not generate any bradykinin, with values similar to background.

**Table 4:** Amount of material required to generate an OD change of 0.2 in the kallikrein generation assay.

Sample	μg
OSCS	6.0
Dextran Sulfate	25.0
Native CS	>100
CS 2.4	34.0
CS 3.0	50.0
CS 3.1	36.0
CS 3.2	23.0
CS 4.0	6.0
CH 4.0 #2	5.0
Heparin	>100

An estimation was made based on the amount of each material required to cause an OD change of 0.2 above baseline, as shown in Fig 1 inset.

### Antithrombin titration

**Fig 3:**
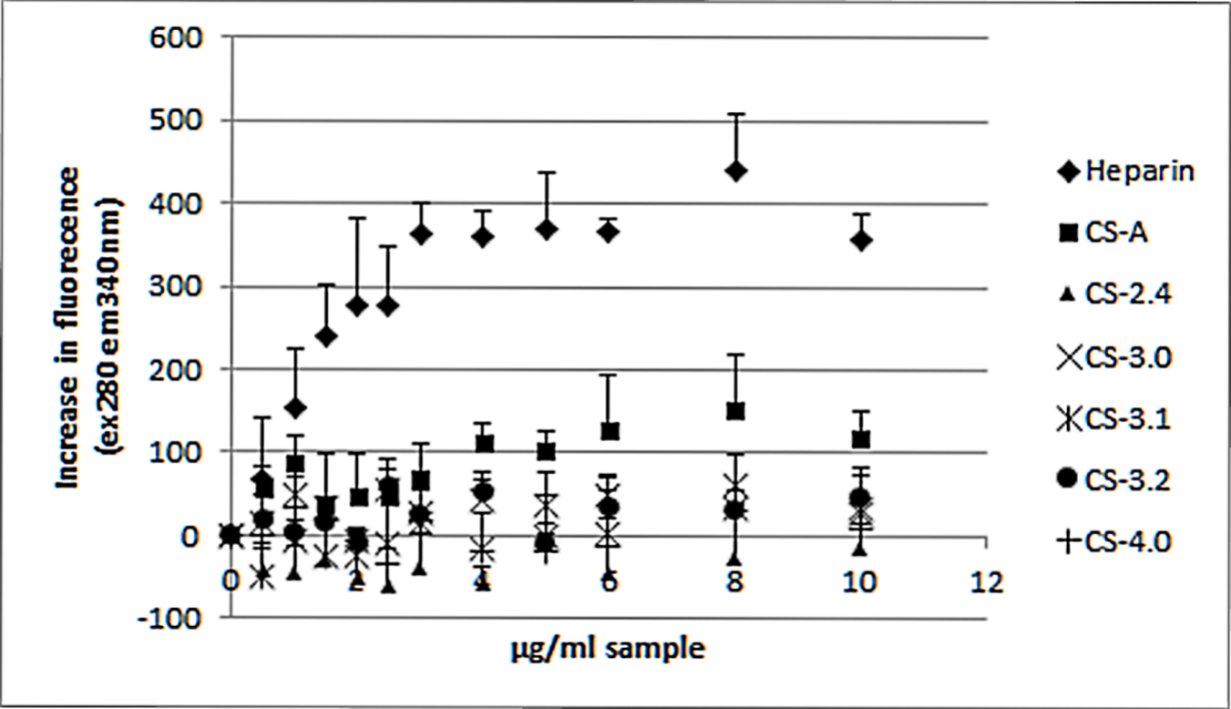
Antithrombin titration response for synthetic OSCS, native chondroitin sulfate and a heparin sample. The increase in fluorescence (ex280nm em340nm) response measured from antithrombin with increasing concentrations of each sample. Error bar is SD, n = 6.

The fluorescence of antithrombin shows a clear rise in signal when increasing levels of heparin are added (Fig 3). Heparin, which binds to antithrombin with high affinity due to the presence of the pentasacchairde sequence, induces a structural change in antithrombin which gives rise to an increase in fluorescence [20]. No oversulfated chondroitin samples were able to elicit a clear increase in fluorescence response, which would indicate that the samples do not bind with any specificity to antithrombin.

## Discussion

Adulteration of heparin with chemically oversulfated chondroitin sulfate was the cause of a contamination issue in 2007/8 [2]. In the current study di-, tri and tetra-sulfated chondroitin sulfates were generated, and NMR and sulfate-to-carboxylate ratio analysis confirmed the structures, with the two tetra-sulfate variants having similar structures to that seen in oversulfated chondroitin sulfate isolated from a sample of the contaminated heparin. In general, the increase in sulfation paralleled a slight increase in molecular weight by both TDA and Broad Standard method, however no increase was seen with CS3.0 to CS4.0 samples. The triple detector method gave higher number average molecular weight, Mn, and weight average molecular weight, Mw, than the broad standard method, and this may be due to the use of an unfractionated heparin sample as the calibrant in the broad standard method which may not provide an accurate calibration for OSCS. Interestingly, the molecular weight observed for the purified OSCS material is lower (Mw 15740 versus 21100 and 21750) than the two synthetic tetra-sulfated versions and is similar to a previous observation [21] for OSCS. This is likely due to differences in the starting CS sample and /or ‘manufacturing’ process, in the same way that unfractionated heparin from different manufacturers possess different molecular weights [22].

The ability of OSCS to act as an anticoagulant via prolongation of clotting times and potentiation of thrombin inhibition in plasma has previously been shown in a limited way [9]. In the present study we have measured the anticoagulant activity of fully and partially sulfated OSCS samples relative to the International Standard for Unfractionated heparin, both in purified and plasma-based assays. This is the first report where the specific activities of variably oversulfated chondroitin sulfate preparations have been determined precisely using validated pharmacopoeial monograph assays and statistical methods. Previous studies have demonstrated anticoagulant activity in plasma and in antithrombin based assays [7–9], but these have been limited as they do not give specific activity, and in the case of plasma assays which showed the presence of anticoagulant activity, but not specifically the mechanisms of this activity. Interestingly all oversulfated samples were found to give statistically valid results, via the multiple dilution analysis method as defined by the European Pharmacopoeia [16]. This is an unusual observation; compounds with such different modes of action compared with the reference heparin standard do not as a rule give valid assays. Unlike heparin, the ability of OSCS samples to bind antithrombin is very limited as shown by antithrombin fluorescence titration. Whilst there is a gradual increase in fluorescence with increasing concentration of material this may be a non-specific interaction.

The addition of extra sulfates to chondroitin sulfate increased the anticoagulant activity in all assays, with some correlation between increasing sulfation and anticoagulant activity. Whilst the addition of sulfates does increase the Mw of the samples, there was no noticeable effect on the anticoagulant activity. Anticoagulant activity measured using plasma (both human and sheep plasma) showed a clear increase based on sulfation, though beyond 3 sulfates per disaccharide there was no difference seen in potency measured by plasma between any of the synthetic samples. In the antithrombin mediated inhibition of activated factor X and thrombin (Table 3), there is a clear increase in activity with additional sulfates, though these activities are very low (<6 IU/mg). This low detectable activity in the presence of antithrombin is non-specific since OSCS is not able to bind to antithrombin with high affinity as it does not contain the essential pentasaccharide sequence (2). The activity was not due to direct inhibition of thrombin or factor Xa.

Several studies have indicated that dermatan sulfate, chondroitin sulfates and other polyanions (such as pentosan polysulfate, dextran sulfate) exhibit their anticoagulant activity independent of antithrombin, and show that this is through heparin cofactor II [11, 23]. Our results in a heparin cofactor II purified assay system, directly provides evidence that OSCS inhibition of thrombin is via interaction with heparin cofactor II. A previous study using surface plasmon resonance has shown that heparin cofactor II binds to OSCS, but this did not show direct potentiation of heparin cofactor II inhibition of thrombin. Dermatan sulfate is considered the main potentiator of HCII *in vivo* [24], and the key sulfate locations are 2-*O*-sulfate on iduronic acid and 4-*O*-sulfate on *N*-acetylgalactosamine which are considered important for binding and thrombin inhibition activity [25, 26]. These positions correspond to 2-*O*-sulfate on glucuronic acid and the 4-*O*-sulfate on *N*-acetylgalactosamine in the over sulfated chondroitin sulfate variants. The increase in 2-*O*-sulfatation on the glucuronic acid from CS2.4 (5%) to CS3.0 (41%) supports this observation. Interestingly the tetra sulfated CS, fully sulfated at positions 2 and 3 on glucuronic acid, gave a lower heparin cofactor II mediated activity than the tri-sulfated CS samples. This lower heparin cofactor II mediated activity in the tetra-sulfated CS could be due to ‘repulsive’ effects, possibly caused by the extra 3-*O*-sulfate on glucuronic acid acting to limit heparin cofactor II binding. Alternatively, the extra sulfate on glucuronic acid may cause some steric hinderance and changes to the conformation of CS [7], thereby preventing the binding of OSCS to heparin co-factor II. In all the assays the chemically prepared tetra-sulfated CS behaves in a similar manner to OSCS purified from contaminated heparin, despite the slightly lower molecular weight.

The reduction in anticoagulant activity as measured by heparin cofactor II when sulfation is increased from 3.0 to 4.0 is not observed with either the sheep or human plasma assays. In both these assays there is no reduction in activity. There are some possible contributory factors which may help to explain these differences. Whilst the activity shown by the antithrombin assays is low, it is known that potentiation of antithrombin inhibition of thrombin is an order of magnitude higher that potentiation of heparin cofactor II inhibition of thrombin [27]. A key difference in the anticoagulant assays is the incubation times that the samples have to potentiate the inhibition of thrombin and factor Xa. In the plasma assay thrombin and factor Xa are generated in situ, whilst for the purified assays thrombin/factor Xa are added and the residual activity measured. The incubation used in heparin cofactor II is much longer (seven minutes) than in the antithrombin dependent assays (two and one minute, factor Xa and thrombin respectively) and the plasma assays (three minutes). Therefore, the contributory effect of heparin cofactor II may be more limited in the plasma assays. Furthermore, heparin-antithrombin can inhibit other coagulation enzymes (factor XIa, factor IXa) [28] which are upstream of factor Xa and thrombin, and may have a role in the plasma assay.

As previously reported [3] OSCS is capable of causing the activation of kallikrein in plasma, though no concentration response was observed. Activation of kallikrein is confirmed by the present study with purified OSCS from adulterated heparin and two chemically prepared tetra sulfate chondroitin sulfates. All three materials were found to give a concentration dependent response, which is similar to a previous report [21] on OSCS from contaminated heparin in a prekallikrein assay. We have clearly shown that increasing the level of sulfation causes an increase in the ability of CS to generate kallikrein. This is likely due to the increase in negative charge by addition of sulfate groups, as in other negatively charged polysaccharides [29, 30] such as dextran sulfate (as shown herein) can also activate prekallikrein. However, charge activation alone may not be sufficient to generate kallikrein since an unadulterated porcine heparin preparation which has similar sulfation to the di-sulfate CS, did not generate any kallikrein. Furthermore, there were some subtle differences in response between CS3.0, CS3.1 and CS3.2 which could indicate that there may be some specific features that influence the activation of kallikrein. Previously it has been shown that OSCS can activate bradykinin [4, 31] and this study under the conditions used the is no bradykinin activity without kallikrein formation. Concentrations of OSCS that generated the same level of kallikrein, also generated the same amount of bradykinin, consistent with prekallikrein activation being the sole mechanism through which OSCS influences bradykinin generation. This suggests that none of the OSCS samples can directly influence formation of bradykinin.

Whilst increasing the level of *O*-sulfation in CS will increase the anti-IIa activity as previously reported [7], given the observed differences between the oversulfated CSs in the kallikrein assay, the results presented here suggest that there may be more subtle structural interactions other than sulfation at play. Production of pure compounds with highly defined patterns of sulfation would be needed to resolve these questions.

## Conclusion

Herein we report the specific anticoagulant activities of differentially oversulfated chondroitin sulfates for the first time. We further show how increased sulfation on chondroitin sulfate results in an increase in activity in several different anticoagulant assay systems. Prekallikrein activation was shown to be both concentration and broadly sulfation dependent.

## Supporting information

S1 File Supporting information Table A and Figures A and B. (DOCX)
