# Supplementary material for: The effect of increasing the sulfation level of chondroitin sulfate on anticoagulant specific activity and activation of the kinin system
Source: PLoS One. 2018 Mar 1;13(3):e0193482. doi: 10.1371/journal.pone.0193482 (PMC5832253; doi:10.1371/journal.pone.0193482)
Supplement: S1 File — (DOCX) [file pone.0193482.s001.docx]

**S1 File - Supporting Information for:** The effect of increasing the sulfation level of chondroitin sulfate on anticoagulant specific activity and activation of the kinin system.

**Table A:** **Table of chemical shifts of methyl signals of the N Acetyl galactosamine** residues in function of the sample degrees of sulfation. The shifts depend on both the degree of sulfation and on sequence effect induced by the heterogeneous sulfations. In a column the colors are indicated in order to help the identification of the corresponding spectra.

| Degree of  ChS sulfation | Spectra identification (colors) | Methyl chemical shifts | | | |
| --- | --- | --- | --- | --- | --- |
|  |  | No extra sulfation  ^1^H (^13^C) ppm | Intermediate sulfation  ^1^H (^13^C) ppm | Intermediate sulfation  ^1^H (^13^C) ppm | Oversulfation  ^1^H (^13^C) ppm |
| CS 1.0 | black | 2.017 (25.34) | - | - | - |
| CS 2.4 | ochre | - | 2.049 | 2.098 | 2.155 |
| CS 3.0 | blue | - | 2.052 (25.64) | 2.091-2.103 | 2.134-2.151 |
| CS 3.1 | green | - | 2.052 (25.67) | 2.091-2.103 | 2.134-2.151 |
| CS 3.2 | brown | - | 2.056 | 2.095-2.105 | 2.137-2.153 |
| CS 4.0 | red | - | - | - | 2.153 (25.72) |

**Figure A: Zoom from 1.9–2.4 p.p.m of the *N*-acetyl region of NMR spectra** of proton (**A**) and HSQC spectra (**B**) of sulfated chondroitins having various degrees of sulfation. Proton spectra profiles are in agreement with literature data^[[1]](#footnote-1)^


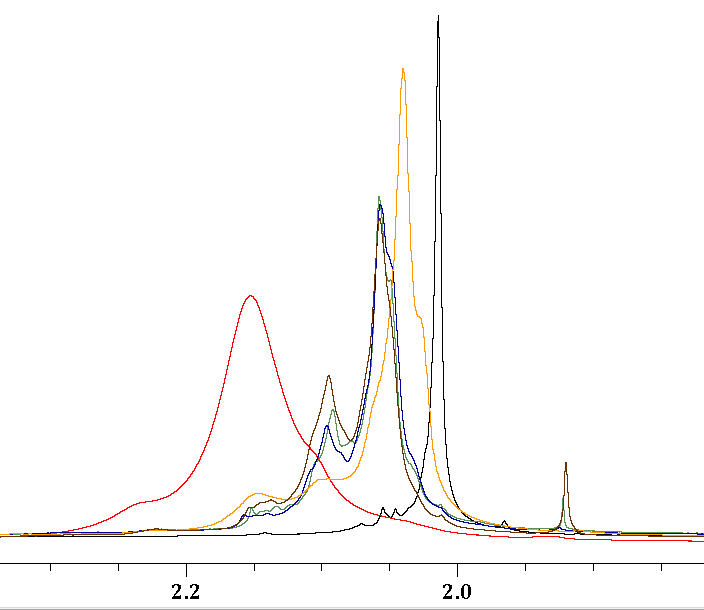


**A**


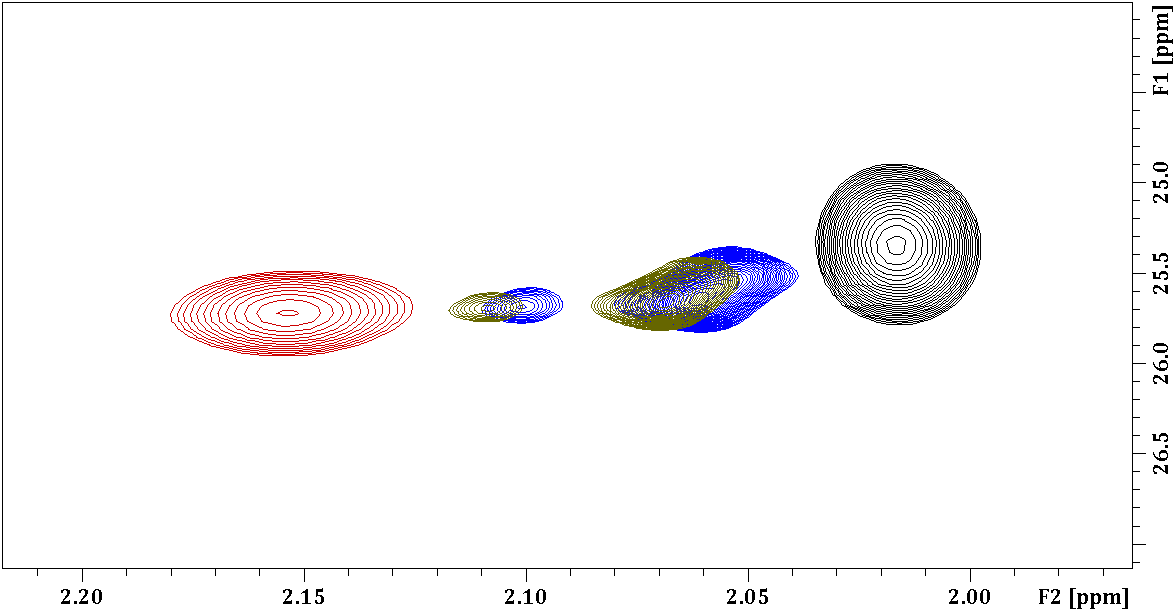

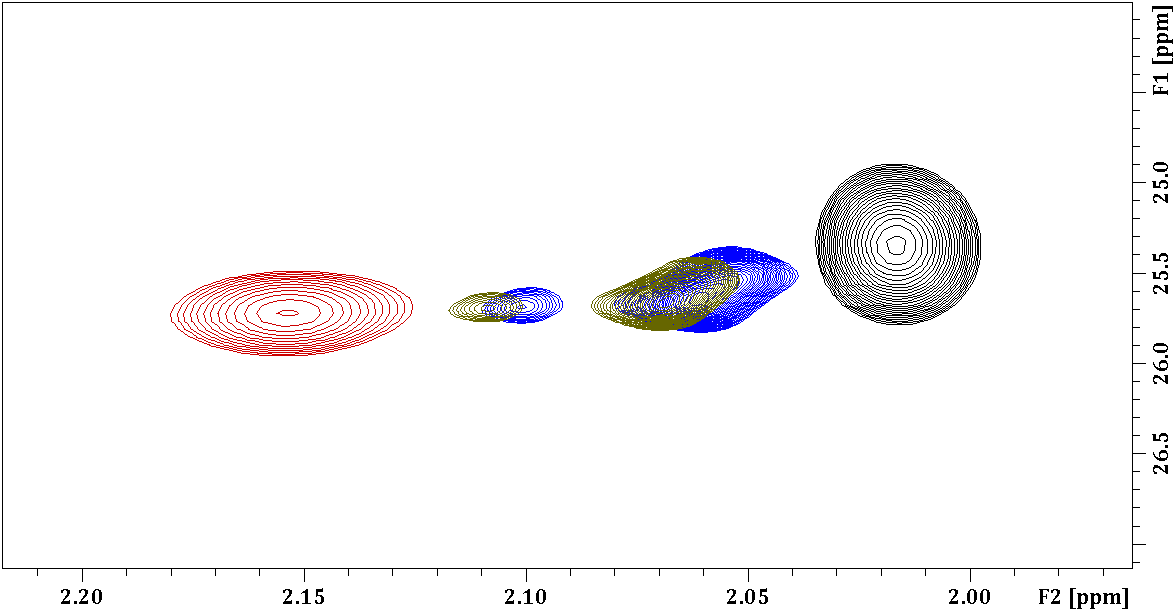


**B**

**Figure B:** **HSQC NMR spectrum of the oversulfated ChS used in the study**. As already evidenced the G5 signals chemical shift strictly depends on the pH of the solution^[[2]](#footnote-2)^. Legend: Galactosamine (A) Glucuronic acid (G); under script residue position substituted by sulfate groups.


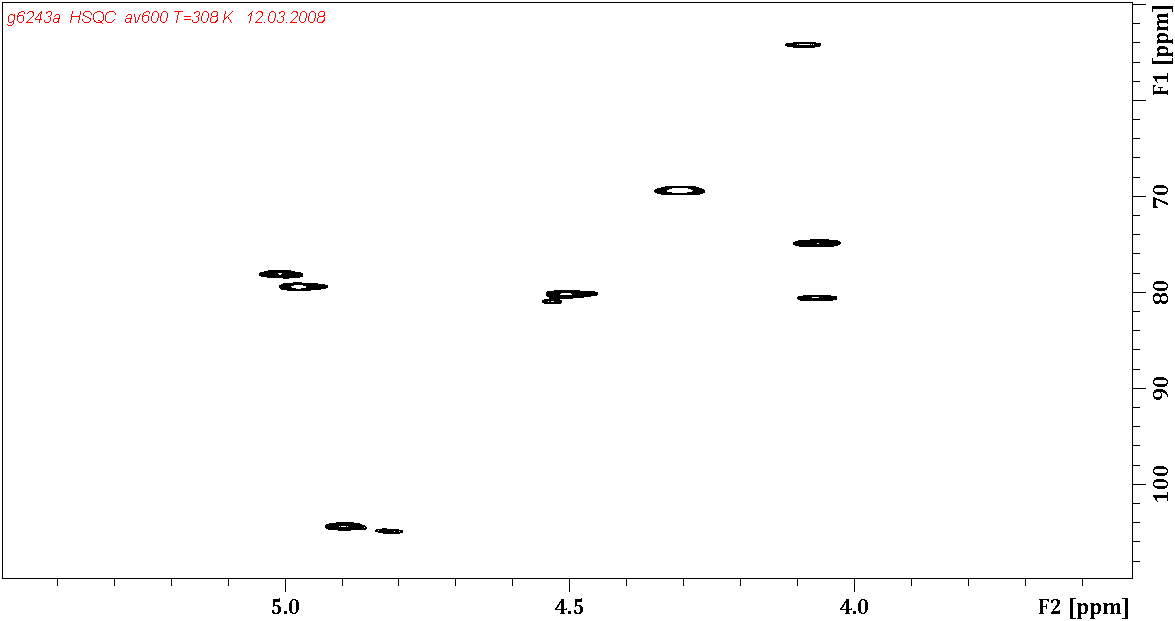


**(G2 + G4) _2,3 S_**

**A4_4,6 S_**

**A2_4,6 S_**

**G3_2,3 S_**

**A1_4,6 S_**

**G1_2,3 S_**

**G5_2,3 S_**

**A6_4,6 S_**

**(A3+A5)_4,6 S_**

**Figure C:** **HSQC anomeric signals assignments of sulfated ChS.** Four samples having different degree of sulfation are overlapped: ChS (black), ChS 3 (blue), ChS 3.1 (green) and ChS 4 (red). Assignments have been done using both model compounds derived from K5 polysaccharide and different NMR correlation techniques such as COSY, TOCSY and HMBC


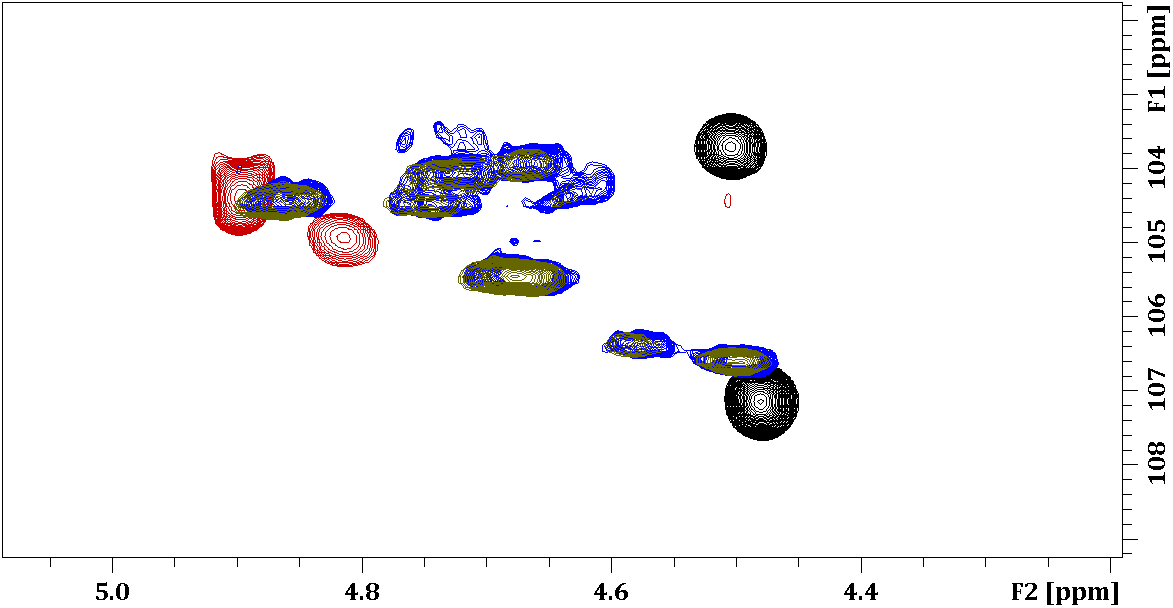


**A**

**G_3S_**

**G**

**G_2S_**

**G_2,3 S_**

**A_xS_**

**A_2,3,4’,6’S_**

**Table B: Kallikrein Generation Assay with variable activation time** – Dextran sulfate (100µg/ml and 25µg/ml) and OSCS (12.5µg/ml and 1.56µg/ml) were incubated with plasma for up to 30 minutes prior to the addition of the kallikrein substrate.

|  |  | Time |  |  |  |  |  |
| --- | --- | --- | --- | --- | --- | --- | --- |
|  |  | 0 | 5 | 10 | 15 | 20 | 30 |
| Dextran Sulfate 100µg/ml | Each point | 0.064 | 0.360 | 0.277 | 0.243 | 0.236 | 0.184 |
|  |  | 0.055 | 0.587 | 0.312 | 0.267 | 0.244 | 0.230 |
|  |  | 0.070 | 0.511 | 0.153 | 0.258 | 0.233 | 0.157 |
|  | **Mean** | **0.063** | **0.486** | **0.247** | **0.256** | **0.238** | **0.190** |
|  | | | | | | | |
| Dextran sulfate 25µg/ml | Each point | 0.046 | 0.318 | 0.222 | 0.202 | 0.204 | 0.165 |
|  |  | 0.047 | 0.301 | 0.176 | 0.156 | 0.096 | 0.107 |
|  |  | 0.054 | 0.161 | 0.051 | 0.049 | 0.049 | 0.054 |
|  | **Mean** | **0.049** | **0.260** | **0.150** | **0.136** | **0.116** | **0.109** |
|  | | | | | | | |
| OSCS 12.5µg/ml | Each point | 0.053 | 0.271 | 0.249 | 0.227 | 0.235 | 0.204 |
|  |  | 0.053 | 0.309 | 0.223 | 0.236 | 0.224 | 0.231 |
|  |  | 0.050 | 0.263 | 0.160 | 0.134 | 0.122 | 0.142 |
|  | **Mean** | **0.052** | **0.281** | **0.211** | **0.199** | **0.193** | **0.192** |
|  | | | | | | | |
| OSCS 1.56µg/ml | Each point | 0.039 | 0.135 | 0.106 | 0.062 | 0.074 | 0.073 |
|  |  | 0.044 | 0.045 | 0.041 | 0.045 | 0.044 | 0.051 |
|  |  | 0.070 | 0.110 | 0.046 | 0.046 | 0.044 | 0.052 |
|  | **Mean** | **0.051** | **0.097** | **0.064** | **0.051** | **0.054** | **0.059** |
|  | | | | | | | |
| Plasma Only | Each point | 0.040 | 0.041 | 0.042 | 0.042 | 0.043 | 0.040 |
|  |  | 0.040 | 0.044 | 0.046 | 0.045 | 0.044 | 0.045 |
|  |  | 0.045 | 0.099 | 0.046 | 0.045 | 0.043 | 0.043 |
|  | **Mean** | **0.042** | **0.061** | **0.045** | **0.044** | **0.043** | **0.043** |

An incubation time of five minutes was found to be optimal for the generation of kallikrein.

**Table C: Anticoagulant potency values for two/three assays used for values in table 3**

|  | IU/mg with calculated 95% Confidence Limits | | | | |
| --- | --- | --- | --- | --- | --- |
| sulfate level / disaccharide | Human Plasma APTT | Sheep Plasma APTT | Antithrombin Anti-Xa | Antithrombin Anti-IIa | Heparin Cofactor II anti-IIa |
| CS 2.4 | 16.3 (16.0 – 16.6)  15.9 (15.6 – 16.1) | 41.1 (39.9 – 42.3)  41.1 (39.5 – 42.8) | 0.78 (0.69 – 0.88)  0.85 (0.66 – 1.08) | 0.86 (0.79 – 0.94)  0.80 (0.71 – 0.90) | 161 (121 – 214)  170 (133 – 218)  138 (115 – 168) |
| CS 3.0 | 51.0 (50.0 – 52.1)  52.9 (52.0 – 53.9) | 131 (126 – 136)  128 (124 – 131) | 1.40 (1.24 – 1.59)  1.31 (1.02 – 1.68) | 2.96 (2.71 – 3.24)  3.34 (2.96 – 3.78) | 823 (620 – 1093)  845 (659 – 1082)  675 (558 – 819) |
| CS 3.1 | 54.5 (53.4 – 55.6)  55.6 (54.6 – 56.6) | 143 (138 – 149)  139 (135 – 143) | 1.61 (1.42 – 1.83)  1.48 (1.15 – 1.89) | 3.99 (3.65 – 4.37)  4.49 (3.97 – 5.09) | 1002 (755 – 1331)  914 (713 – 1171)  861 (710 – 1043) |
| CS 3.2 | 51.0 (50.0 - 52.1)  53.0 (52.1 – 54.0) | 140 (135 – 146)  136 (132 – 140) | 2.04 (1.80 – 2.32)  1.67 (1.30 – 2.15) | 3.49 (3.18 – 3.82)  4.07 (3.59 – 4.63) | 867 (654 – 1151)  1038 (811 – 1332)  836 (690 – 1012) |
| CS 4.0 | 48.5 (47.5 – 49.5)  47.7 (46.8 – 48.6) | 138 (132 – 143)  136 (132 – 140) | 5.20 (4.58 – 5.90)  5.73 (4.48 – 7.39) | 4.94 (4.51 – 5.40)  4.11 (3.63 – 4.65) | 417 (314 – 554)  352 (275 – 451)  248 (204 – 301) |
| CS 4.0 (#2) | 50.2 (49.2 – 51.2)  48.9 (48.0 – 49.8) | 142 (136 – 148)  142 (138 – 146) | 5.92 (5.22 – 6.71)  5.86 (4.55 – 7.49) | 3.92 (3.58 – 4.29)  3.48 (3.07 – 3.94) | 341 (256 – 452)  288 (224 – 369)  203 (167 – 246) |
| OSCS isolated from Contaminated Heparin | 56.5 (54.4 – 58.7)  54.8 (53.0 – 56.6) | 171 (162 – 179)  186 (178 – 194) | 5.37 (5.02 – 5.74)  6.05 (5.74 – 6.37) | 5.00 (4.73 – 5.29)  4.79 (4.55 – 5.04) | 200 (181 – 221)  174 (159 – 191) |
| Chondroitin 4 sulfate | 0.40 (0.37 – 0.43)  0.42 (0.40 – 0.43) | 0.71 (0.19 – 1.02)  0.74 (0.35 – 0.99) | 0.59 (0.53 – 0.65)  0.56 (0.51 – 0.61) | 0.34 (0.33 – 0.36  0.35 (0.33 – 0.36) | 1.94 (1.74 – 2.16)  1.89 (1.69 – 2.14) |
| Heparin sample | 237 (224 – 250)  184 (174 – 194)  212 (202 – 221) | 225 (195 – 274)  219 (208 – 231)  232 (222 – 243) | 206 (196 – 217)  229 (208 – 252)  218 (207 – 231) | 197 (190 – 205)  202 (193 – 212)  233 (218 – 245) | 241 (209 – 272)  244 (212 – 280)  237 (204 – 276) |

*Potency values were estimated against the 5^th^ International Standard for unfractionated heparin 97/578 (NIBSC, South Mimms, UK) in accordance to European Pharmacopoeia Chapter 5.3*

**Table D:** **Kallikrein Generation Assay** – individual data points used to create figure 1

|  | OSCS from contaminated Heparin | | | |  |  |
| --- | --- | --- | --- | --- | --- | --- |
| µg/ml | Run 1 | Run 2 | Run 3 | Run 4 | Run 5 | Run 6 |
| 100 | 0.601 | 0.644 | 0.573 | 0.798 | 0.507 | 0.759 |
| 50 | 0.502 | 0.672 | 0.542 | 0.733 | 0.457 | 0.611 |
| 25 | 0.423 | 0.447 | 0.428 | 0.536 | 0.420 | 0.465 |
| 12.5 | 0.330 | 0.333 | 0.339 | 0.347 | 0.353 | 0.347 |
| 6.25 | 0.247 | 0.255 | 0.227 | 0.148 | 0.286 | 0.263 |
| 3.125 | 0.146 | 0.186 | 0.110 | 0.049 | 0.229 | 0.208 |
| 1.5625 | 0.123 | 0.147 | 0.047 | 0.048 | 0.176 | 0.153 |
| 0.78125 | 0.075 | 0.088 | 0.045 | 0.048 | 0.069 | 0.111 |
|  |  |  |  |  |  |  |
|  | Dextran Sulfate | |  |  |  |  |
| µg/ml | Run 1 | Run 2 | Run 3 | Run 4 | Run 5 | Run 6 |
| 100 | 0.612 | 0.690 | 0.628 | 0.448 | 0.720 | 0.652 |
| 50 | 0.401 | 0.420 | 0.376 | 0.319 | 0.504 | 0.418 |
| 25 | 0.204 | 0.268 | 0.247 | 0.259 | 0.292 | 0.241 |
| 12.5 | 0.060 | 0.129 | 0.179 | 0.145 | 0.077 | 0.172 |
| 6.25 | 0.070 | 0.101 | 0.085 | 0.070 | 0.050 | 0.058 |
| 3.125 | 0.049 | 0.067 | 0.067 | 0.051 | 0.048 | 0.060 |
| 1.5625 | 0.048 | 0.046 | 0.050 | 0.050 | 0.047 | 0.039 |
| 0.78125 | 0.049 | 0.047 | 0.046 | 0.051 | 0.046 | 0.047 |
|  |  |  |  |  |  |  |
|  | Chondrontin Sulfate (Native CS) | | | |  |  |
| µg/ml | Run 1 | Run 2 | Run 3 | Run 4 | Run 5 | Run 6 |
| 100 | 0.059 | 0.078 | 0.066 | 0.067 | 0.064 | 0.067 |
| 50 | 0.055 | 0.059 | 0.061 | 0.060 | 0.060 | 0.060 |
| 25 | 0.054 | 0.055 | 0.052 | 0.054 | 0.056 | 0.054 |
| 12.5 | 0.054 | 0.055 | 0.053 | 0.055 | 0.056 | 0.053 |
| 6.25 | 0.050 | 0.053 | 0.049 | 0.051 | 0.054 | 0.054 |
| 3.125 | 0.051 | 0.053 | 0.053 | 0.050 | 0.054 | 0.052 |
| 1.5625 | 0.049 | 0.052 | 0.051 | 0.054 | 0.052 | 0.052 |
| 0.78125 | 0.047 | 0.056 | 0.049 | 0.052 | 0.050 | 0.049 |
|  |  |  |  |  |  |  |
|  | 2.4 Sulfate Chondroitin Sulfate | | | |  |  |
| µg/ml | Run 1 | Run 2 | Run 3 | Run 4 | Run 5 | Run 6 |
| 100 | 0.599 | 0.659 | 0.482 | 0.406 | 0.630 | 0.427 |
| 50 | 0.369 | 0.346 | 0.299 | 0.318 | 0.400 | 0.325 |
| 25 | 0.257 | 0.129 | 0.161 | 0.261 | 0.192 | 0.233 |
| 12.5 | 0.179 | 0.122 | 0.058 | 0.181 | 0.120 | 0.192 |
| 6.25 | 0.068 | 0.053 | 0.059 | 0.070 | 0.051 | 0.114 |
| 3.125 | 0.051 | 0.051 | 0.053 | 0.049 | 0.050 | 0.070 |
| 1.5625 | 0.045 | 0.040 | 0.049 | 0.056 | 0.061 | 0.055 |
| 0.78125 | 0.046 | 0.043 | 0.048 | 0.045 | 0.046 | 0.044 |
|  |  |  |  |  |  |  |
|  | 3.0 Sulfate Chondroitin Sulfate | | | |  |  |
| µg/ml | Run 1 | Run 2 | Run 3 | Run 4 | Run 5 | Run 6 |
| 100 | 0.558 | 0.121 | 0.469 | 0.492 | 0.364 | 0.412 |
| 50 | 0.295 | 0.160 | 0.262 | 0.258 | 0.277 | 0.255 |
| 25 | 0.181 | 0.194 | 0.215 | 0.220 | 0.182 | 0.176 |
| 12.5 | 0.145 | 0.112 | 0.145 | 0.151 | 0.115 | 0.126 |
| 6.25 | 0.090 | 0.115 | 0.151 | 0.166 | 0.051 | 0.074 |
| 3.125 | 0.109 | 0.072 | 0.147 | 0.115 | 0.049 | 0.071 |
| 1.5625 | 0.075 | 0.071 | 0.086 | 0.060 | 0.048 | 0.049 |
| 0.78125 | 0.048 | 0.054 | 0.062 | 0.048 | 0.047 | 0.050 |
|  |  |  |  |  |  |  |
|  | 3.1 Sulfate Chondroitin Sulfate | | | |  |  |
| µg/ml | Run 1 | Run 2 | Run 3 | Run 4 | Run 5 | Run 6 |
| 100 | 0.578 | 0.533 | 0.550 | 0.215 | 0.707 | 0.613 |
| 50 | 0.315 | 0.307 | 0.322 | 0.171 | 0.429 | 0.318 |
| 25 | 0.212 | 0.223 | 0.204 | 0.162 | 0.258 | 0.224 |
| 12.5 | 0.125 | 0.173 | 0.156 | 0.057 | 0.113 | 0.110 |
| 6.25 | 0.088 | 0.121 | 0.101 | 0.077 | 0.048 | 0.050 |
| 3.125 | 0.072 | 0.106 | 0.094 | 0.052 | 0.049 | 0.058 |
| 1.5625 | 0.050 | 0.056 | 0.060 | 0.051 | 0.049 | 0.049 |
| 0.78125 | 0.048 | 0.053 | 0.051 | 0.051 | 0.047 | 0.054 |
|  |  |  |  |  |  |  |
|  | 3.2 Sulfate Chondroitin Sulfate | | | | |  |
| µg/ml | Run 1 | Run 2 | Run 3 | Run 4 | Run 5 | Run 6 |
| 100 | 0.610 | 0.645 | 0.507 | 0.399 | 0.761 | 0.803 |
| 50 | 0.374 | 0.353 | 0.266 | 0.309 | 0.483 | 0.454 |
| 25 | 0.253 | 0.190 | 0.149 | 0.272 | 0.410 | 0.325 |
| 12.5 | 0.212 | 0.146 | 0.056 | 0.234 | 0.249 | 0.234 |
| 6.25 | 0.095 | 0.046 | 0.070 | 0.139 | 0.174 | 0.195 |
| 3.125 | 0.065 | 0.039 | 0.049 | 0.049 | 0.102 | 0.175 |
| 1.5625 | 0.042 | 0.030 | 0.049 | 0.045 | 0.061 | 0.085 |
| 0.78125 | 0.048 | 0.049 | 0.047 | 0.045 | 0.046 | 0.054 |
|  |  |  |  |  |  |  |
|  | 4.0 Sulfate Chondroitin Sulfate | | | |  |  |
| µg/ml | Run 1 | Run 2 | Run 3 | Run 4 | Run 5 | Run 6 |
| 100 | 0.788 | 0.893 | 0.857 | 0.517 | 0.587 | 0.724 |
| 50 | 0.491 | 0.626 | 0.617 | 0.346 | 0.441 | 0.545 |
| 25 | 0.414 | 0.457 | 0.442 | 0.297 | 0.407 | 0.401 |
| 12.5 | 0.347 | 0.345 | 0.322 | 0.287 | 0.362 | 0.312 |
| 6.25 | 0.245 | 0.242 | 0.213 | 0.252 | 0.257 | 0.233 |
| 3.125 | 0.184 | 0.150 | 0.127 | 0.206 | 0.073 | 0.119 |
| 1.5625 | 0.068 | 0.097 | 0.105 | 0.129 | 0.079 | 0.077 |
| 0.78125 | 0.062 | 0.047 | 0.057 | 0.050 | 0.047 | 0.065 |
|  |  |  |  |  |  |  |
|  | 4.0 Sulfate Chondroitin Sulfate #2 | | | |  |  |
| µg/ml | Run 1 | Run 2 | Run 3 | Run 4 | Run 5 | Run 6 |
| 100 | 0.473 | 0.554 | 0.607 | 0.375 | 0.474 | 0.526 |
| 50 | 0.455 | 0.528 | 0.635 | 0.314 | 0.435 | 0.521 |
| 25 | 0.425 | 0.476 | 0.512 | 0.299 | 0.405 | 0.466 |
| 12.5 | 0.387 | 0.459 | 0.408 | 0.291 | 0.366 | 0.372 |
| 6.25 | 0.295 | 0.334 | 0.279 | 0.280 | 0.296 | 0.272 |
| 3.125 | 0.275 | 0.198 | 0.161 | 0.220 | 0.176 | 0.157 |
| 1.5625 | 0.159 | 0.050 | 0.116 | 0.166 | 0.116 | 0.103 |
| 0.78125 | 0.114 | 0.047 | 0.044 | 0.079 | 0.058 | 0.064 |
|  |  |  |  |  |  |  |
|  | Unfractionated Heparin | | |  |  |  |
|  | Run 1 | Run 2 | Run 3 | Run 4 | Run 5 | Run 6 |
| 100 | 0.044 | 0.076 | 0.071 | 0.047 | 0.053 | 0.048 |
| 50 | 0.043 | 0.062 | 0.056 | 0.059 | 0.057 | 0.050 |
| 25 | 0.042 | 0.058 | 0.045 | 0.060 | 0.052 | 0.048 |
| 12.5 | 0.041 | 0.054 | 0.052 | 0.047 | 0.047 | 0.044 |
| 6.25 | 0.039 | 0.045 | 0.044 | 0.042 | 0.046 | 0.046 |
| 3.125 | 0.039 | 0.047 | 0.044 | 0.043 | 0.046 | 0.045 |
| 1.5625 | 0.044 | 0.043 | 0.042 | 0.041 | 0.039 | 0.039 |
|  |  |  |  |  |  |  |
|  | Run 1 | Run 2 | Run 3 | Run 4 | Run 5 | Run 6 |
| Blank | 0.465 | 0.047 | 0.045 | 0.046 | 0.044 | 0.054 |

**Table E:** **Kallikrein Generation Assay** – Raw optical density data points used for figure 2A

| Sample | µg material | Assay 1 | Assay 2 | Assay 3 | Assay 4 | Assay 5 | Assay 6 |
| --- | --- | --- | --- | --- | --- | --- | --- |
| OSCS | 6 | 0.321 | 0.269 | 0.311 | 0.252 | 0.308 | 0.367 |
| DX | 25 | 0.381 | 0.172 | 0.266 | 0.305 | 0.313 | 0.500 |
| CSA | 100 | 0.062 | 0.065 | 0.059 | 0.058 | 0.059 | 0.050 |
| CS 2.4 | 34 | 0.409 | 0.310 | 0.309 | 0.289 | 0.303 | 0.523 |
| CS 3.0 | 50 | 0.422 | 0.238 | 0.277 | 0.294 | 0.285 | 0.513 |
| CS 3.1 | 36 | 0.369 | 0.222 | 0.267 | 0.265 | 0.288 | 0.444 |
| CS 3.2 | 23 | 0.313 | 0.282 | 0.267 | 0.266 | 0.289 | 0.430 |
| CS 4.0 | 6 | 0.272 | 0.270 | 0.345 | 0.258 | 0.329 | 0.339 |
| CS 4.0 #2 | 5 | 0.315 | 0.257 | 0.358 | 0.265 | 0.326 | 0.349 |
| UFH | 100 | 0.057 | 0.064 | 0.061 | 0.061 | 0.061 | 0.062 |
| Blank (plasma only) | | 0.050 | 0.050 | 0.045 | 0.046 | 0.044 | 0.046 |

**Table F:** **Bradykinin Generation Assay** – Individual ng/ml bradykinin values used figure 2B

|  |  | ng/ml bradykinin | | | | | |
| --- | --- | --- | --- | --- | --- | --- | --- |
|  | µg material | Set 1 | Set 2 | Set 3 | Set 4 | Set 5 | Set 6 |
| OSCS | 6 | 24.16 | 29.57 | 32.78 | 34.64 | 32.80 | 28.04 |
| DX | 25 | 40.62 | 31.80 | 18.00 | 39.21 | 36.10 | 36.78 |
| CSA | 100 | 0.46 | 0.22 | 0.76 | 0.50 | 0.52 | 0.54 |
| CS 2.4 | 34 | 28.62 | 16.77 | 31.58 | 34.91 | 28.09 | 34.14 |
| CS 3.0 | 50 | 38.61 | 29.11 | 33.36 | 33.50 | 32.46 | 31.95 |
| CS 3.1 | 36 | 34.43 | 30.61 | 32.33 | 32.52 | 24.31 | 39.00 |
| CS 3.2 | 23 | 32.14 | 28.00 | 31.34 | 37.40 | 34.38 | 32.90 |
| CS 4.0 | 6 | 27.77 | 23.18 | 30.08 | 32.65 | 32.86 | 26.88 |
| CS 4.0 #2 | 5 | 28.97 | 16.80 | 30.25 | 35.60 | 25.39 | 37.28 |
| UFH | 100 | 0.59 | 0.25 | 0.38 | 0.36 | 0.15 | 0.02 |
| Plasma only | | 0.48 | 0.20 | 0.53 | 0.29 | 0.48 | 0.10 |

**Table G: Antithrombin Titration Assay –** Individual points used for figure 3

| **OSCS from contaminated heparin** | | | | | |
| --- | --- | --- | --- | --- | --- |
| µg/ml | µg in assay | Run 1 | Run 2 | Run 3 | Run 4 |
| 0 | 0 | 0 | 0 | 0 | 0 |
| 5 | 0.5 | -16.18 | -17.17 | -10.37 | -12.33 |
| 10 | 1 | 10.88 | -5.74 | 17.94 | -1.16 |
| 15 | 1.5 | 53.36 | -5.42 | -7.96 | 43.40 |
| 20 | 2 | 60.73 | 88.15 | -12.22 | -8.86 |
| 25 | 2.5 | 7.21 | 32.39 | -33.06 | 8.90 |
| 30 | 3 | 60.10 | 132.99 | 23.15 | 55.60 |
| 40 | 4 | 117.50 | 59.21 | 99.13 | 28.38 |
| 50 | 5 | 93.04 | 107.25 | 184.50 | 250.83 |
| 60 | 6 | 159.56 | 114.88 | 40.25 | 121.07 |
| 80 | 8 | 195.72 | 123.90 | 127.80 | 102.07 |
| 100 | 10 | 119.11 | 163.11 | 79.09 | 35.13 |
| **Chondroitin Sulfate A** | | | | | |
| µg/ml | µg in assay | Run 1 | Run 2 | Run 3 | Run 4 |
| 0 | 0 | 0 | 0 | 0 | 0 |
| 5 | 0.5 | 92.18 | 43.54 | 19.37 | 68.014 |
| 10 | 1 | 143.05 | 51.62 | 80.07 | 70.248 |
| 15 | 1.5 | 137.62 | 34.47 | -6.19 | -17.748 |
| 20 | 2 | 104.27 | 85.26 | -22.41 | 27.349 |
| 25 | 2.5 | 55.73 | 89.00 | 75.85 | -25.844 |
| 30 | 3 | 115.40 | 101.85 | 17.14 | 41.476 |
| 40 | 4 | 141.98 | 120.95 | 102.27 | 81.635 |
| 50 | 5 | 144.60 | 95.28 | 80.50 | 86.999 |
| 60 | 6 | 146.36 | 166.75 | 12.56 | 181.907 |
| 80 | 8 | 213.89 | 137.96 | 207.58 | 49.842 |
| 100 | 10 | 129.25 | 79.85 | 162.07 | 107.884 |
| **Chondroitin sulfate 2.4** | | | | | |
| µg/ml | µg in assay | Run 1 | Run 2 | Run 3 | Run 4 |
| 0 | 0 | 0 | 0 | 0 | 0 |
| 5 | 0.5 | -113.79 | -31.46 | -12.75 | -8.60 |
| 10 | 1 | -100.21 | -43.74 | -31.97 | -7.76 |
| 15 | 1.5 | -79.05 | -1.28 | -45.23 | 11.27 |
| 20 | 2 | -76.09 | -36.91 | -30.00 | -57.48 |
| 25 | 2.5 | -94.57 | -31.38 | -32.68 | -78.92 |
| 30 | 3 | -92.50 | -34.61 | -31.11 | 2.32 |
| 40 | 4 | -122.79 | -30.39 | -76.91 | -2.83 |
| 50 | 5 | -57.39 | -24.76 | 12.23 | 27.02 |
| 60 | 6 | -85.41 | -47.83 | -16.21 | -28.75 |
| 80 | 8 | -90.48 | -25.74 | -31.22 | 44.86 |
| 100 | 10 | -105.92 | 6.40 | -4.40 | 45.16 |
| **Chondroitin sulfate 3.0** | | | | | |
| µg/ml | µg in assay | Run 1 | Run 2 | Run 3 | Run 4 |
| 0 | 0 | 0 | 0 | 0 | 0 |
| 5 | 0.5 | -44.16 | -25.50 | 50.63 | 83.58 |
| 10 | 1 | 29.26 | 12.94 | 100.67 | 54.82 |
| 15 | 1.5 | 30.48 | 18.32 | 51.88 | 31.48 |
| 20 | 2 | -28.22 | -15.04 | 16.15 | -0.90 |
| 25 | 2.5 | 48.49 | 49.37 | 53.25 | 65.72 |
| 30 | 3 | 8.31 | 39.02 | -16.63 | 75.65 |
| 40 | 4 | 14.73 | 46.51 | 30.85 | 82.36 |
| 50 | 5 | -0.26 | -0.55 | 68.85 | 82.57 |
| 60 | 6 | 23.35 | 38.13 | 57.72 | 82.90 |
| 80 | 8 | 39.75 | 7.35 | 28.08 | 61.80 |
| 100 | 10 | 57.38 | 9.21 | 7.94 | 33.67 |
| **Chondroitin sulfate 3.1** | | | | | |
| µg/ml | µg in assay | Run 1 | Run 2 | Run 3 | Run 4 |
| 0 | 0 | 0 | 0 | 0 | 0 |
| 5 | 0.5 | -110.42 | -51.77 | -10.27 | -21.58 |
| 10 | 1 | -53.52 | -4.33 | -81.07 | 118.34 |
| 15 | 1.5 | -89.73 | 9.87 | -71.75 | 57.32 |
| 20 | 2 | -56.75 | -31.39 | -39.78 | 27.79 |
| 25 | 2.5 | -108.22 | 0.60 | -12.71 | 77.93 |
| 30 | 3 | -58.39 | 52.77 | -13.80 | 74.83 |
| 40 | 4 | -51.03 | -40.73 | -30.06 | 57.62 |
| 50 | 5 | -71.33 | 0.53 | 3.41 | 66.86 |
| 60 | 6 | -76.39 | 20.07 | -2.73 | 67.69 |
| 80 | 8 | 16.43 | 91.12 | 27.05 | 105.70 |
| 100 | 10 | -6.61 | 85.59 | -4.52 | 60.80 |
| **Chondroitin sulfate 3.2** | | | | | |
| µg/ml | µg in assay | Run 1 | Run 2 | Run 3 | Run 4 |
| 0 | 0 | 0 | 0 | 0 | 0 |
| 5 | 0.5 | 67.95 | 21.50 | 4.15 | -18.89 |
| 10 | 1 | -1.25 | -7.34 | 28.88 | -5.90 |
| 15 | 1.5 | 32.67 | 1.07 | 36.84 | -4.54 |
| 20 | 2 | -11.27 | -28.07 | -0.34 | 5.78 |
| 25 | 2.5 | 81.24 | 54.74 | 27.12 | 70.33 |
| 30 | 3 | 75.03 | 25.33 | 7.95 | -1.42 |
| 40 | 4 | 88.53 | 56.36 | 28.49 | 36.37 |
| 50 | 5 | -5.11 | -9.61 | -25.47 | 4.06 |
| 60 | 6 | 93.99 | 32.62 | 13.90 | 4.82 |
| 80 | 8 | 31.57 | 30.16 | 41.12 | 29.83 |
| 100 | 10 | 99.22 | 31.07 | 52.72 | 4.00 |
| **Chondroitin Sulfate 4.0** | | | | | |
| µg/ml | µg in assay | Run 1 | Run 2 | Run 3 | Run 4 |
| 0 | 0 | 0 | 0 | 0 | 0 |
| 5 | 0.5 | 18.96 | 7.04 | -11.51 | -12.83 |
| 10 | 1 | 80.77 | -37.61 | -3.26 | 90.76 |
| 15 | 1.5 | 16.50 | 18.08 | -3.88 | 42.35 |
| 20 | 2 | 21.11 | 16.27 | -56.16 | -3.11 |
| 25 | 2.5 | -39.02 | -5.03 | -96.40 | 0.77 |
| 30 | 3 | 13.24 | 52.29 | 12.41 | -9.98 |
| 40 | 4 | -23.31 | -34.13 | -30.82 | 7.98 |
| 50 | 5 | 0.34 | -81.20 | -13.24 | 17.80 |
| 60 | 6 | -9.86 | -56.18 | 1.32 | 19.25 |
| 80 | 8 | 19.46 | 19.53 | 38.16 | 45.84 |
| 100 | 10 | 40.79 | -47.48 | 35.13 | 6.75 |
| **Unfractionated Heparin** | | | | | |
| µg/ml | µg in assay | Run 1 | Run 2 | Run 3 | Run 4 |
| 0 | 0 | 0 | 0 | 0 | 0 |
| 5 | 0.5 | 63.29 | 182.57 | 42.15 | -21.37 |
| 10 | 1 | 245.70 | 188.44 | 127.39 | 53.40 |
| 15 | 1.5 | 321.61 | 277.08 | 194.57 | 163.13 |
| 20 | 2 | 286.79 | 430.81 | 249.43 | 141.12 |
| 25 | 2.5 | 374.48 | 292.99 | 263.99 | 174.07 |
| 30 | 3 | 369.53 | 376.17 | 403.19 | 306.65 |
| 40 | 4 | 385.42 | 394.53 | 346.93 | 311.27 |
| 50 | 5 | 276.90 | 443.55 | 425.48 | 337.56 |
| 60 | 6 | 379.27 | 360.70 | 384.41 | 345.80 |
| 80 | 8 | 493.85 | 495.13 | 441.95 | 331.78 |
| 100 | 10 | 325.30 | 409.23 | 357.83 | 332.61 |

1. Guerrini, M. *et al. correspondence on Nat. Biotechnol.* **28**, (3) 207-211, 2010 [↑](#footnote-ref-1)
2. Guerrini, M. *et al. Nat. Biotechnol.* **26**, 669–675 (2008).

   Oversulfated chondroitin sulfate is a contaminant in heparin associated with adverse clinical events Marco Guerrini, Daniela Beccati, Zachary Shriver, Annamaria Naggi, Karthik Viswanathan, Antonella Bisio, Ishan Capila, Jonathan C Lansing, Sara Guglieri, Blair Fraser, Ali Al-Hakim, Nur Sibel Gunay, Zhenqing Zhang, Luke Robinson, Lucinda Buhse, Moheb Nasr, Janet Woodcock, Robert Langer, Ganesh Venkataraman, Robert J Linhardt, Benito Casu, Giangiacomo Torri & Ram Sasisekharan

   Nature Biotechnology **26**, 669–675 (2008) [↑](#footnote-ref-2)
